# A Review of Non-Surgical Pain Management in Osteoarthritis

**DOI:** 10.7759/cureus.10829

**Published:** 2020-10-06

**Authors:** Neha Shah, Shaheen Jadidi, Prempreet Bajaj

**Affiliations:** 1 Department of Physical Medicine and Rehabilitation, Northwestern Medicine at Marianjoy Rehabilitation Hospital, Wheaton, USA; 2 Family Medicine, McGaw Medical Center of Northwestern University, Chicago, USA; 3 Department of Orthopedic Surgery and Rehabilitation, Loyola University Medical Center, Maywood, USA

**Keywords:** osteoarthritis, degenerative joint disease, pain management, sports medicine, non-surgical, orthopedics, preventative medicine, exercise, radiofrequency ablation

## Abstract

Osteoarthritis is a prominent cause of disability in older adults, especially with an increasingly obese and aging population. Clinical management of pain secondary to osteoarthritis should focus on education and self-management with exercise and weight management as a priority. Surgical intervention should only be considered once conservative measures have failed. This review provides a clinical update on the pathogenesis, diagnosis, and management of osteoarthritis.

## Introduction and background

Osteoarthritis is a degenerative joint disease involving the cartilage and surrounding tissues of a joint. The cartilage itself is devoid of innervation and not the source of pain associated with osteoarthritis. Rather the wearing away of the cartilage exposes the highly innervated periosteum to damage, and this leads to the perception of “joint pain” [1]. Osteoarthritis is the most common form of arthritis and a leading cause of disability and debility in the United States [2]. Disease progression is generally slow, and over time it causes damage to the articular cartilage, remodeling of bone, ligament laxity, and osteophyte formation to name a few [3]. As such, osteoarthritis can be thought of as a disease of the whole joint. Pain is often the chief complaint and dominant symptom of osteoarthritis, and there is evidence that it should be addressed within a biopsychosocial model that is individualized to the patient [4].

Osteoarthritis also represents an extremely large health burden with significant implications not only in health care but socioeconomically as well [5]. Increasing obesity levels combined with a growing aging population globally has caused osteoarthritis to be a significant and growing issue. Knee osteoarthritis is the most common site of osteoarthritis, followed by the hand and hip, and is found to be present in 19.2%-27.8% of adults above 45 years and 37.4% of adults above 60 years [6]. This figure is even larger worldwide with a recent global analysis approximating that 85% of osteoarthritis cases being located in the knee [7]. While increases in life span and body mass index are thought to contribute to the significant rise in knee osteoarthritis in the post-industrial era, those factors alone do not explain this change [8]. Regardless of the cause, the burden of osteoarthritis leads to a decrease in the quality of life. The presence of chronic knee pain has been found to be a strong predictor of future disability and dependency in older adults [9]. 

There is evidence that among this large and growing population of patients suffering from osteoarthritis, many of them do not receive optimal management [10]. This review will present recent updates in the understanding of osteoarthritis and appropriate management of associated symptoms.

## Review

Assessing the Patient

Osteoarthritis is a diagnosis made clinically based off of symptoms including pain, functional limitations, and morning stiffness among others. A brief physical examination can reveal many of the appropriate diagnostic criteria such as restricted or painful movement, crepitus, and joint-line tenderness, and is enough to make a diagnosis [11]. It has been shown that the rate of radiographic osteoarthritis is much higher than that of symptomatic arthritis [12], but plain radiographs should be considered if the presentation is atypical or other diagnoses are suspected. Women are more at risk of developing osteoarthritis. Between the ages of 50 and 75 years, the risk for hand and knee osteoarthritis appears to increase much more rapidly in women when compared to men [5]. For knee osteoarthritis, strong risk factors besides age, obesity, and female sex include previous knee injury as well as malalignment of the knee [13] and should be assessed, especially, in patients who were or are athletes. In addition, knee extensor muscle strength should be evaluated as this has also been shown to be a risk factor for knee osteoarthritis [14]. For hip osteoarthritis, the joint should be assessed for deformities such as acetabular dysplasia or cam deformity as this has been shown to be a risk factor [15]. In terms of social risk factors, work that involves heavy lifting has been shown to increase the risk of both hip and knee osteoarthritis, and work that involves a lot of kneeling is associated with increased risk of knee osteoarthritis [16]. Assessment should include a complete history and physical examination, with particular attention given to how symptoms are affecting quality of life, including function, mood, relationships, leisure activities, and sleep as this facilitates shared decision-making, which has been shown to ultimately improve outcomes [17].

The most common chief complaint in patients with osteoarthritis is pain. Other symptoms include crepitus, reduced range of motion, morning stiffness, swelling, muscle weakness, and joint instability [18]. Of note, comorbidities such as cardiovascular disease and diabetes have been shown to be predictive of faster deterioration and progression of pain [19]. The pain in osteoarthritis is typically brought on with weight-bearing activity and is as such predictable, especially in early stages. Once the disease progresses the symptoms of pain tend to become more severe and unpredictable, both of which have been shown to be extremely strong indicators of patient’s finding their pain unacceptable [20]. 

X-ray and MRI imaging have been shown to reveal only moderate associations between the presence of pain and structural osteoarthritis [21]. However, this association becomes much stronger in patients with very frequent pain symptoms [22]. Of note, specific findings including synovitis and bone marrow lesions appear to contribute more to pain than overall structural change to pain in osteoarthritis [23].

Increased activity of peripheral nociceptors caused by progressive tissue injury and inflammation in the affected joint is the primary nociceptive pain mechanism in osteoarthritis; however, neuropathic and central pain mechanisms seem to be present in a large proportion of patients with osteoarthritis [24]. Central pain mechanisms include loss of descending antinociceptive pathways as well as enhanced descending pain pathway activity [25]. Neuropathic pain appears to arise from nerve changes in peripheral nervous system or spinal cord as well as structural changes in joint innervation itself [26].

Non-Surgical Management 

Self-management, exercise, weight loss (if overweight or obese), walking aids (if needed), and education are widely accepted as first-line treatment for osteoarthritis and should be initiated as early as possible in the disease process [27]. Patient education should focus on the importance of regular physical activity and exercise as well as appropriate weight management in addition to information about how these conservative measures can potentially avoid surgery and improve quality of life [28,29]. Individualized exercise regimens, particularly strength training and aerobic exercises, have been shown to be extremely effective in improving joint motion and decreasing pain [30].

Initial pharmacological management in osteoarthritis includes non-steroidal anti-inflammatory drugs (NSAIDs) and acetaminophen. However, a recent meta-analysis has concluded that acetaminophen is not an effective first-line treatment due to its small area of effect, while oral NSAIDs are effective in terms of clinical improvement of function and pain, although both have safety concerns [31]. Because safety is an important consideration when choosing an appropriate NSAID, it is preferable to restrict usage to only as needed at the smallest effective dose. Of note, a 2018 meta-analysis revealed that topical NSAIDs were effective for pain relief in osteoarthritis with no serious gastrointestinal or renal side effects [32]. In addition, some guidelines advise caregivers to consider duloxetine for pain refractory to initial pharmacological management. It is thought that the anxiolytic effects coupled with central pain inhibition help address neuropathic and central pain mechanisms contributing to osteoarthritis [33]. A meta-analysis published in 2015 concluded that celecoxib, glucosamine, chondroitin, and the combination of glucosamine and chondroitin help relieve joint pain when compared with placebo, but only glucosamine plus chondroitin showed clinically significant improvement from baseline function and therefore should also be considered for non-surgical management [34]. It should be noted, however, that health authorities and health insurers currently do not cover these preparations.

Patients that do not or no longer respond to oral or topical analgesics should begin intra-articular corticosteroid injections. Patients with evidence of joint inflammation have been shown to respond better to corticosteroid injection than those without, and a large body of evidence suggests that intra-articular corticosteroid injections are much more effective in patients with severe pain compared to those with less pain [27]. Alternatives such as hyaluronic acid injections have been shown to have beneficial effects such as intra-articular lubrication, anti-inflammatory, analgesic, and chondroprotective effects, among others [35]; however, there are inconsistent findings on clinical studies and a high cost associated with this treatment, which has led to the most recent Osteoarthritis Research Society and American College of Rheumatology guidelines to neither recommend nor discourage the use of this treatment [36].

Comparisons between non-opiates and opiates for the relief of pain in osteoarthritis have shown a greater effect with non-opiates as well as increased risk of adverse effects from opiates [37]. This in addition to the high risk of addictive behavior and overdosage has led to opposition to prescribing opioids to patients with osteoarthritis in the medical community [38]. In addition, a 2019 Cochrane Systematic Review showed that tramadol alone and in combination with acetaminophen had no important benefit on pain reduction compared to placebo control [39]. 

The literature around the innervation of the knee has shown marked variability in the distribution of the nerve supply, and while there is general consensus that the nerves involved are branches of the femoral, sciatic, and obturator, there has been disagreement within the scientific community about exactly which nerves supply the knee capsule and what are the best targets for radiofrequency ablation (RFA) [40,41]. For the purposes of this paper, we will follow the studies by Gardner [40] and Kennedy [42], which are the basis of most anatomy textbooks, and simplify the nerves by splitting them into two groups: an anterior and a posterior group of nerves. The posterior group of afferent nerves originates from the sciatic nerve (primarily its tibial branch) and a variable amount by branches from the posterior branch of the obturator nerve, which form a plexus with the popliteal vessels. The largest and most consistent nerve supplying the knee is the posterior articular branch of the posterior tibial nerve. The tibial nerve remains in the posterior compartment of the leg and gives off the superomedial (SM) and inferomedial (IM) genicular nerves. The posterior group supplies the innervation to the posterior capsule, the external part of the menisci, the anterior and posterior cruciate ligaments, and may reach as far as the infrapatellar (IP) fat pad. The anterior group includes the afferent nerves of the femoral (the branches to vastus medialis, vastus lateralis, and vastus intermedius) and the common peroneal nerve with two articular nerves (the lateral articular and recurrent peroneal nerves) [40,41]. The common peroneal splits off the sciatic nerve but passes into the anterior compartment and contributes to the superior lateral (SL) genicular nerve. The anterior group supplies the anteromedial and anterolateral capsules as well as the associated ligaments. 

The specific nerves targeted most commonly by radiofrequency neurotomy are the three genicular nerves mentioned: superolateral genicular branch from the common peroneal nerve, the SM, and IM genicular branches from the tibial nerve. The technique for targeting these nerves was popularized by Choi et al. [43] who found significant improvement in pain at three months in the neurotomy patients versus control patients who received a sham procedure with a block. However, despite the popularity of this approach, the anatomical basis and reliability of the bony landmarks described in the study were confusing and created some controversy with the accuracy of the targets being questioned. Part of this was due to a study being performed at the same time by Ikeuchi et al. where they compared RFA of knee sensory nerves at two different sites (the medial retinacular nerve and the IP branch of the saphenous nerve) to a nerve block [44], with similarly positive results at three months.

The result of this complex innervation of the knee has been the development of multiple approaches being described in different studies. In a review of current literature conducted by Jamison and Cohen, they reviewed nine clinical trials which all not only demonstrated significant benefits in reduction of pain and improvement in function but also mentioned the wide range of procedural targets. These targets included the SM, IM, and SL genicular nerves in combination; the saphenous nerve; the sciatic nerve; the IP genicular nerve; the IP and SM genicular nerves in combination; the femoral, tibial, saphenous, and peripatellar plexus in combination; and the intra-articular joint space [45].

On April 13, 2017, the Federal Drug Administration approved cooled RFA as a treatment option for chronic moderate to severe knee pain caused by osteoarthritis. The approval of this approach to treat osteoarthritis pain in the knee was based on a randomized, double-blinded study funded by the device manufacturer (Halyard Health, now known as Avanos, Georgia, USA), which compared the ablation of the three genicular nerves technique (popularized by Choi et al.) via this new delivery system to a single intra-articular steroid injection into the affected knee. This study showed 74.1% of cooled RFA patients reported greater than 50% pain relief at six months versus 16.2% of intra-articular steroid injection patients [46]. Prior to this development, the treatment of pain from late-stage osteoarthritis of the knee was essentially limited to surgery. As a result, in 2003, 402,100 people underwent total knee arthroplasties with a projection of as many as 3.4 million replacements annually by 2030 [47]. However, not all patients are candidates for surgery due to age or comorbidities, and after having failed conservative treatment options such as bracing, physical therapy, medical management, and steroid or hyaluronic acid derivative injections, there were no other treatment options. The results of this study were similar to a 2016 study performed by Sari et al., which found genicular nerve radiofrequency neurotomy to be superior to an intra-articular steroid injection into the knee at one and three months [48].

As the use of genicular nerve RFA has expanded, so too has its applications, and we now see it being used for chronic pain that persists after total knee replacement [49]. A 2017 randomized study by McCormick et al. looked at the validity of performing a prognostic block prior to cooled RFA and found no difference in outcomes between patients who had a positive block compared to patients that did not have a block; 58.6% of nerve block patients and 64% of non-nerve block patients had at least 50% improvement in pain at six months [50]. While this again shows significant improvement in pain as a result of RFA, there is still a large percentage of patients that did not have a good response to the procedure. The reasoning for these “failed” procedures is mixed. Choi et al. hypothesized that the patients who did not respond to RFA occurred due to the variable innervation patterns of the knee [43]. The Jamison et al. review examined poor predictors of outcome based primarily on a review of literature from lumbar facet and sacroiliac joint RFA studies. Certain patient characteristics such as opioid use, age > 65 years, higher baseline pain, and history of depression were thought to play a role, as were procedural factors. These procedural factors included the type of RFA used (cooled RFA was found to create a larger lesion that continuous RFA); within the continuous RFA and pulsed RFA groups, larger probes and duration of treatment were found to have larger lesions. The larger lesions were thought to relate to some of the better outcome seen in cooled RFA versus continuous or pulsed RFA, but results have been mixed [45]. 

## Conclusions

The global population will be confronted with an increasing burden of osteoarthritis in the coming years, and addressing the disease process early in its course will likely vastly improve outcomes. In order to accomplish this, preventive measures beyond promoting weight loss and exercise should be investigated, as well as interventions addressing risk factors for osteoarthritis and specific guidelines for early diagnostic criteria and management. In addition, different phenotypes of pain are an emerging topic that is relevant to osteoarthritis and its management. As our understanding of these phenotypes increase, providers should use this knowledge to individualize treatment for osteoarthritis. Such targeted treatment can help with adherence and prevention of progression of osteoarthritis.
