# Correction: A Review of Non-Surgical Pain Management in Osteoarthritis

**DOI:** 10.7759/cureus.c45

**Published:** 2021-07-14

**Authors:** Neha Shah, Shaheen Jadidi, Prempreet Bajaj

**Affiliations:** 1 Department of Physical Medicine and Rehabilitation, Northwestern Medicine at Marianjoy Rehabilitation Hospital, Wheaton, USA; 2 Family Medicine, McGaw Medical Center of Northwestern University, Chicago, USA; 3 Department of Orthopedic Surgery and Rehabilitation, Loyola University Medical Center, Maywood, USA

Due to a misunderstanding between authors, two of the original authors were not listed on this article at the time of submission and publication. All three authors have discussed this and determined that adding the two missing authors is the best solution. The two new authors are:

**Neha Shah** Department of Physical Medicine and Rehabilitation, Northwestern Medicine at Marianjoy Rehabilitation Hospital, Wheaton, USA

**Prempreet Bajaj** Department of Orthopedic Surgery and Rehabilitation, Loyola University Medical Center, Maywood, USA

